# Mental health status of pregnant women during COVID-19 in healthcare centers of Iran: A cross-sectional study

**DOI:** 10.1371/journal.pone.0294850

**Published:** 2023-11-28

**Authors:** Masoumeh Sayahi, Maryam Nikbina, Azam Jahangirimehr, Barat Barati

**Affiliations:** 1 Department of Midwifery, Shoushtar Faculty of Medical Sciences, Shoushtar, Iran; 2 Department of Public Health, Shoushtar Faculty of Medical Sciences, Shoushtar, Iran; 3 Department of Radiologic Technology, Shoushtar Faculty of Medical Sciences, Shoushtar, Iran; Iranian Institute for Health Sciences Research, ISLAMIC REPUBLIC OF IRAN

## Abstract

**Background and objective:**

The COVID-19 pandemic impacted every single aspect of life. In addition to being a public health emergency, the COVID-19 outbreak impacted the mental health of individuals, especially pregnant women. This study aimed to examine the mental health status of pregnant women and also the effect of sociodemographic factors on their mental health status during COVID-19 in healthcare centers of Iran.

**Methods:**

This cross-sectional, analytical-descriptive study was conducted among pregnant women referring to healthcare centers in Shoushtar, Iran, in 2021. Multistage cluster sampling was used to select participants. Data were collected using the General Health Questionnaire-28 (GHQ-28). Data were analyzed using SPSS software version 22. The Pearson correlation coefficient was used to examine the association between quantitative variables. A generalized linear model (GLM) was applied to estimate the effect of independent variables on the dependent variable (mental health).

**Results:**

A total of 197 participants with a mean ± SD age of 27.85 ± 6.37 years took part in this study. The total mean score of mental health was estimated at 17.47±8.20. The highest mean ± SD score was, respectively, related to social dysfunction (6.63 ± 2.86), anxiety and insomnia (5.28 ± 3.53), and somatic symptoms (4.17 ± 3.27). Mental health disorder was significantly correlated with participants’ age (R = .223, P = .00), number of pregnancy (gravida) (R = .272, P = .00), number of births (para) (R = 0.272, P = .00), and number of abortions (R = .172, P = .015). About 80% of pregnant women did not reveal impaired mental health conditions or psychological distress, while 19.3% showed scores that indicate probable mental health conditions.

**Conclusion:**

Social dysfunction was the most common mental health problem among pregnant women. It is necessary to pay more attention to the mental health status of pregnant women during a pandemic. Interventions such as practical strategies to promote social support and improve pregnant women’s mental health during pregnancy are highly important.

## Introduction

COVID-19 was a novel infectious disease caused by a new strain of the severe acute respiratory syndrome coronavirus, SARS-CoV-2. In December 2019, COVID-19 was initially identified in Wuhan City, Hubei Province, China, and quickly disseminated to all corners of the globe [1–3].

Governments have implemented strict measures like quarantine, travel restrictions, and curfews due to the high transmission rate of the virus among people and the insufficient understanding of this new virus. Prolonged social and physical distancing and uncertainty about the future have led to distress and affected people’s mental health and well-being [4, 5].

COVID-19 lead to psychological distress and increased mental health problems, such as depressive symptoms, anxiety, stress, insomnia, anger, and fear [6]. As per the reports from the WHO, signs of depression and anxiety have increased worldwide due to the COVID-19 pandemic [7]. These symptoms are higher among vulnerable groups [8–10]. Meanwhile, pregnant women as vulnerable individuals, their mental health is a public health priority and requires special attention during the pandemic [6, 11–14].

Pregnancy is a unique and natural physiological process in women’s lives and is associated with significant changes in mental and physical health [15]. Mental health issues are a significant global health concern that affects various populations, particularly pregnant women [16]. The risk for anxiety and depression during pregnancy is higher, and pregnant women are at higher risk for depressive disorder with peripartum onset [17]. Worldwide 10% of pregnant women and 13% of mothers experience a mental disorder, primarily depression. These rates are higher in low and middle income countries (LMICs) [18]. Anxiety and depression in pregnancy can lead to increased toxicity, nausea, vomiting, the risk of preterm birth, lower birth weight, and a lower Apgar score [19].

Pregnant women are at a higher risk of contracting COVID-19 due to their vulnerability to other diseases caused by highly pathogenic coronaviruses such as SARS and MERS, as well as the potential impact on their pregnancy [20–22]. It has been shown that anxiety in pregnant women is 3–4 times greater than in the general population during COVID-19. A study has shown that the prevalence of anxiety and depression among pregnant women has risen during the COVID-19 pandemic [23].

A study in China examined the psychiatric consequences of COVID-19 and illustrated moderate to severe depressive symptoms (16.5%), moderate to severe anxiety symptoms (28.8%), and moderate to severe stress levels (8.1%). They also showed females are more prone to develop symptoms such as depression, anxiety, insomnia, and stress [24]. Moreover, a study by Saccone in Italy demonstrated that the COVID-19 outbreak had a moderate to severe psychological impact on pregnant women [25]. A systematic review reported the prevalence of stress was the highest psychological impact of COVID-19 among pregnant and lactating women [26].

Given that the COVID-19 pandemic is a universal stressor experienced worldwide, the mental health impact of the pandemic can differ based on interpersonal and contextual factors [27]. Contextual factors like socioeconomic status can increase susceptibility to the negative impacts of COVID-19 specific stressors [28]. It has been shown women who experience sociodemographic risk factors are more likely to be susceptible to psychopathology [29–31].

Assessment of the negative effects of COVID-19 during pregnancy is crucial to identify the possible interventions in order to prevent the harmful and negative effects on both maternal and fetal outcomes. To date, a very few studies have examined the mental health status of pregnant women during COVID-19 in Iran and the effect of sociodemographic factors on their mental health status.

Most of the studies have examined the relationship between sexual function, quality of life and mental health in pregnant women [32, 33], obstetrics healthcare providers’ mental health and quality of life [34], and health anxiety and related factors [35, 36] among pregnant women during the COVID-19. Therefore, this study aimed to examine mental health status of pregnant women and also the effect of sociodemographic factors on their mental health status during COVID-19 in health care centers of Iran.

## Materials and methods

### Study design and setting

This cross-sectional, analytical-descriptive study was conducted in primary healthcare centers in Shoushtar, Iran, in 2021. Shoushtar City is located in the north part of Khuzestan province in the southwest of Iran. The county has 9 primary health centers affiliated with the Shoushtar Faculty of Medical Sciences.

### Study participants and sampling

The study population included 197 pregnant women referring to primary healthcare centers in Shoushtar, Iran. A multi-stage cluster sampling method was used to select participants. There are nine health centers in the city, of which four were randomly selected. Pregnant women were systematically randomly selected from each center using their medical record numbers.

### Inclusion and exclusion criteria

The inclusion criteria were as follows: being a pregnant woman, willing to participate in the study, residing in the recruitment area, negative for COVID-19 infection, absence of previously detected chronic and debilitating diseases (e.g., diabetes, cancer, etc.). Those who used sedative medications, those with a history of mental health disorders, those who were divorced or separated, and those who had lost their loved ones during the last six months were excluded from the study.

### Data collection tools

A questionnaire consisting of two part was used for data collection. The first part was related to demographic and socioeconomic questions (n = 20). The second part was the General Health Questionnaire-28 (GHQ-28). The GHQ-28 was developed by Goldberg and Hillier in 1979 [37]. The GHQ-28 is a self-administered screening instrument for psychiatric disorders in nonclinical populations [37]. The questionnaire has been translated into 38 languages and studied in various cultural settings [38]. The Persian questionnaire version was also developed [39].

GHQ-28 consisted of four subscales consisting of 7 items in each case, which are labelled somatic symptoms (items 1–7), anxiety and insomnia (items 8–14), social dysfunction (items 15–21), and severe depression (items 22–28). Responses to all questions were scored on a 4-point Likert scale. The minimum score is 0, and the maximum is 84. Higher scores indicate higher levels of distress. Scores of 0–22 indicate non-psychiatric, 23–40 mild psychiatric, 41–60 moderate psychiatric, and scores 61–84 indicate severe psychiatric. Each item of the mental health problems was scored as follows: scores of 0–6 were considered as non-psychiatric, 7–11 were considered as a mild psychiatric, 12–16 were considered as moderate psychiatric, and 17–21 were considered as severe psychiatric. The internal consistency validity of GHQ using Cronbach’s alpha was 0.88. The sensitivity of this test has been reported to be 0.84 with a specificity of 0.82 [40].

### Procedures

Data were collected by one of the trained researchers (MN) from March to July, 2021. Questionnaires were completed by the pregnant women. Those who were literate filled in the questionnaire, and those who were illiterate the questions were read to them by a midwife, and they responded accordingly. All women were informed about the aim of the study, and gave their verbal and written consent before being included in it. Owing to the pandemic, each participant was contacted by telephone to verify that they fulfilled all the inclusion criteria, and thereafterresearcher arranged a mutually convenient time to interview those who met the inclusion criteria and agreed to participate. Participants were interviewed in a private room within the healthcare centers. Each interview lasted ∼20 min (range: 7–46 min).

### Ethical considerations

This study was approved by the Ethics Committee of the Shoushtar Faculty of Medical Sciences (Reference No: IR.SHOUSHTAR.REC.1399.040), and conducted in accordance with the ethical principles of the Declaration of Helsinki. The study was conducted after obtaining ethical approval. Permission was obtained from the authorities of the study site prior to the study. The participants were informed that their participation was voluntary and that their answers would be anonymous and confidential. Informed written consent was obtained from study participants whose ages were 18 years and more. For participants who were below the age of 18 years, written consent was obtained from their parents. All methods were performed in accordance with relevant guidelines and regulations that must be considered in research where humans are involved.

#### Data analysis

Data were analyzed using SPSS _v22.0_. For descriptive statistics, frequency and percentage, median, means, and standard deviation were used. The normality of the numeric variables was tested using the Kolmogorov-Smirnov test. The Pearson correlation coefficient was used to examine the association between quantitative variables. A generalized linear model (GLM) was used to compare the association between the variables in the presence of other contextual factors on mothers’ mental health. In this study, the dependent variable was mental health; independent quantitative variables (e.g. women’s age, gestational age, the number of births, the number of abortions, and BMI) were included as covariates in the model; independent qualitative variables (e.g. women’s education status, spouse’s education status, women’s employment status, spouse’s employment status, and income level) were included as fixed effects in the model. All tests of associations were carried out at a level of significance of < .05.

## Results

A total of 197 pregnant women were included in the study. Most of the pregnant women (82.7%) and their spouses (81.7%) had a high-school diploma or below. 91.9% of women were housewives, and 47.2% reported their husbands were self-employed. The income level of most participants was more than 15 million Rials (Table 1).

**Table 1:** The socio-demographic characteristics of participants.

Variables	Modes	Frequency (%)
**Pregnant women’s education status**	**High school diploma and below**	163 (82.7)
	**Academic degree**	34 (17.3)
**Spouse’s education status**	**High school diploma and below**	161 (81.7)
	**Academic degree**	36 (18.3)
**Pregnant women’s employment status**	**Employed**	8 (4)
	**Worker**	3 (1.5)
	**Self-employed**	5 (2.5)
	**Housewife**	181 (91.9)
**Spouse’s employment status**	**Employed**	20 (10.2)
	**Worker**	47 (23.9)
	**Farmer**	22 (11.2)
	**Self-employed**	97 (49.2)
	**Unemployed**	11 (5.6)
**Income level**	**Less than 5 million Rials**	20 (10.2)
	**5–10 million Rials**	34 (17.3)
	**10–15 million Rials**	62 (31.5)
	**More than 15 million Rials**	81 (41.1)

In this study, the age range of participants was between 14 and 45 years, with a mean ±SD age of 27.85 ± 6.37 years. The mean ± SD of women’s Body Mass Index (BMI) was 26.93 ± 4.52 Kg/m^2^, ranging from 17.41 to 31 kg/ m^2^. The median gestational age was 20 (interquartile range (IQR)  =  19–30), ranging from 18 to 40. The median gravida was 2 (IQR: 1–3), with a range from 1 to 8. The median parity per woman was 1 (IQR  =  0–2), with a range from 0 to 5 (Table 2).

**Table 2:** Participants’ pregnancy information.

Variable	Median (IQR)	Minimum	Maximum
**Gestational age**	20 (19–30)	18	40
**Gravida**	2 (1–3)	1	8
**Parity**	1 (0–2)	0	5
**Abortion**	0 (0–1)	0	3

In this study, the total mean ± SD score of mental health among pregnant women was estimated at 17.47±8.20, ranging from 3 to 52. The highest mean ± SD score of mental health was related to the social dysfunction subscale at 6.63 ± 2.86 (ranging from 0 to 21), followed by somatic symptoms at 5.28 ± 3.53 (ranging from 0 to 21) and anxiety and insomnia at 4.17 ± 3.27 (ranging from 0 to 16).

As shown in Table 3, 80.7% of pregnant women did not reveal impaired mental health conditions or psychological distress, while 19.3% showed scores that indicate probable mental health conditions. More than half of the participants (54.8%) had mild social dysfunction.

**Table 3:** Mental health scores in pregnant mothers.

Subscales	non- psychiatric n (%)	mild- psychiatric n (%)	Moderate—psychiatric n (%)	Severe-psychiatric n (%)
**Somatic symptoms**	144 (73.1)	40 (20.3)	11 (5.6)	2 (1)
**Anxiety and insomnia**	156 (79.2)	34 (17.3)	7 (3.6)	0 (0)
**Social dysfunction**	81 (41.1)	108 (54.8)	7 (3.6)	1 (0.5)
**Severe depression**	189 (95.9)	4 (2)	2 (1)	2 (1)
**The total score of mental health**	159 (80.7)	32 (16.3)	6 (3)	0 (0)

Table 4 represents the correlation between socio-demographic variables and sub-scales of mental health disorders. Mental health disorder was significantly correlated with women’s age (R = .223, P = .00), gravida (R = .272, P = .00), parity (R = 0.267, P = .00), and number of abortion (R = .172, P = .015). The results also showed a significant correlation between education status of women and social dysfunction (P >.05).

**Table 4:** The correlation between demographic characteristics and mental health subscales.

Variable	Somatic symptoms	Anxiety and insomnia	Social dysfunction	Severe depression	Total
**Women’s age**	(R = .177, P = .013*)	(R = .139, P = .052*)	(R = .165, P = .021*)	(R = .089, P = .213)	(R = .223, P = .002**)
**Gestational age**	(R = .069, P = .334)	(R = .098, P = .171)	(R = .38, P = .595)	(R = .137, P = .055*)	(R = .001, P = .993)
**Gravida**	(R = .153, P = .02*)	(R = .210, P = .003**)	(R = .274, P = .002**)	(R = .179, P = .012*)	(R = .272, P = .000**)
**Parity**	(R = .187, P = .08)	(R = .166, P = .02*)	(R = .252, P = .000**)	(R = .124, P = .083)	(R = .267, P = .000**)
**Number of abortion**	(R = .028, P = .697)	(R = .198, P = .005*)	(R = .081, P = .256)	(R = .177, P = .013*)	(R = .172, P = .015)
**Women’s education status**	(R = .026, P = .721)	(R = .129, P = .07)	(R = .187, P = .009**)	(R = .126, P = .078)	(R = .013, P = .86)
**Spouse’s education status**	(R = .082, P = .250)	(R = .072, P = .315)	(R = .17, P = .017*)	(R = .069, P = .335)	(R = .054, P = .451)
**Income level**	(R = .122, P = .089)	(R = .029, P = .684)	(R = .116, P = .106)	(R = .017, P = .814)	(R = .096, P = .181)
**BMI**	(R = .063, P = .378)	(R = .080, P = .265)	(R = .001, P = .985)	(R = .052, P = .464)	(R = .058, P = .420)

Pearson correlation test. Analysis significant at < .05 level.

*Significant at p < 0.05 level.

**Significant at p < 0.01 level.

The results of GLM are shown in Table 5. Mental health disorder was significantly associated with women’s age (P = 0.00) and gravida (P = 0.04). Multigravida women and older women were more likely to experience mental health disorders.

**Table 5:** Factors affecting pregnant women’s mental health.

Variables	SS	df/F	P-Value
**Women’s age**	465.90	1/7.99	0.005**
**Gravida**	217.40	1/3.73	0.045*
**Paity**	107.41	1/1.84	0.176
**Number of abortion**	70.45	1/1.20	0.273

*Significant at p < 0.05 level.

**Significant at p < 0.01 level.

## Discussion

This study was designed to examine the mental health status of pregnant women during COVID-19. While pregnancy can be a stressful experience for many women [41], the COVID-19 pandemic has introduced an additional layer of concern regarding its potential impact on the health of mothers and the delivery of their babies [42].

In this study, only 19.8% of women reported having had mental health problems during pregnancy. Previous research in other countries showed that pregnant women during the COVID-19 had more psychopathological symptoms, such as depression and anxiety, than those who were pregnant before the COVID-19 pandemic [26, 42–46]. Since COVID-19 was a novel virus, there was not enough epidemiological information early on about how easily this virus can spread between people [47]; therefore, people were more likely to experience stress.

COVID-19 has had mental/emotional, and social implications for pregnant and postpartum women who have been physically separated from family members, relatives, and society [26]. In the present study, the highest mean score was related to social dysfunction, somatic symptoms, anxiety and insomnia, and severe depression, respectively. Social dysfunction among pregnant and lactating women during COVID-19 was also reported in a previous study [48].

Social dysfunction can be attributed to the lack of social support from family, partner or peers. A study by Liu et al., demonstrated that social support from family was associated with low levels of depression, whereas support from partners or friends was not associated with any mental health problems [49].

Previous studies showed increased perceived social support and support effectiveness were associated with lower mental health symptoms, and were protective factors against depression and anxiety [50–52]. A review study demonstrated social support and being engaged in regular physical activities can be protective factors to buffer the effects of the pandemic on maternal mental health [53].

COVID-19 has put a lot of pressure on the healthcare system, and there have been many changes in the way of providing health care [54]. COVID-19 disrupted maternity services and increased risks of maternal morbidity and mortality during this period [55]. Studies have shown that pregnant women were less likely to seek support for mental health on their own due to fear of being shamed, embarrassed, socially isolated, and stigmatized [56–59].

A lack of social support, disappointment and a lack of trust were identified as significant barriers to seeking mental health services among women with postpartum depression and pregnant women [60, 61]. It seems the interpersonal factors are important in help-seeking. A Chinese study reported that participants who received high levels of social support during the COVID-19 pandemic were more likely to seek psychological help [60]. Previous studies have demonstrated there was a significant association between social support and positive attitudes toward seeking mental health services [62, 63], and perceived need for psychological help [64].

Health professionals play a crucial role in ameliorating the emotional and mental well-being of pregnant women [65, 66], and have a high responsibility of supporting pregnant women achieve their mental health needs [67]. Support from health professionals causes pregnant women more likely to seek help for their mental health issues [67].

Anxiety and insomnia ranked third in this study. During a pandemic, anxiety is anticipated to be high. The findings of this study are in keeping with previous studies, which illustrated that pregnant and lactating women had insomnia during COVID-19 [68, 69]. Increased anxiety and depression levels were also reported among pregnant women during COVID-19 [70–72]. According to a study conducted in Spain, insomnia was found to be associated with increased depressive symptoms and stress among pregnant women [43]. Furthermore, some studies demonstrated that levels of insomnia increased in the general population during COVID-19 [73–76].

The age of pregnant women in the current study was found to be correlated with their mental health. This result was also confirmed by GLM. Older women were more likely to have higher somatic symptoms, anxiety, insomnia, and social dysfunction. It seems that increasing age is associated with a decrease in a mother’s physical and mental capacity; therefore, it makes one prone to various diseases, including mental and/ or psychological diseases. According to a prior study, age can exacerbate feelings of isolation and marginalization during pregnancy [77].

In this study, the number of abortion was significantly correlated with mental health problems (e.g. anxiety, insomnia, and severe depression). A study by Mascio demonstrated that abortion during COVID-19 affected the mental health of pregnant women and caused problems such as anxiety, sleep disorder, physical problems, and depression among these groups [78]. Laisk et al. found that pregnant women may experience increased depression and stress as a result of early pregnancy loss [79].

In this study, pregnant women’s mental health was significantly correlated with the gravida and parity. Women who were multigravida and multiparous were more likely to experience anxiety, insomnia, and social dysfunction. Further, the results of GLM showed a significant association between gravida and mental health disorders in pregnant women. An Indonesian study demonstrated that subjects with higher gravida had higher odds of anxiety levels [80].

Moreover, we found a significant correlation between the women’s education status and social dysfunction and a significant correlation between the spousal education status and social dysfunction. This is supported by a previous study that showed that high knowledge levels could lead to a decline in the anxiety level of pregnant women [70]. Women with higher education levels may use different information resources and engage in a more detailed search about COVID-19. Pregnant women’s anxiety levels may be impacted by a greater understanding of the potential adverse impacts of the disease on the health of both the mother and the fetus [70]. Our results were not in line with a previous study that demonstrated a high level of spousal education was associated with reduced symptoms of stress and anxiety in pregnant women [81].

In this study, income level was not correlated with the mental health of pregnant women. Previous studies reported depression adversely affected the health outcomes of low-income mothers [82, 83]. Also, Thayer et al. in 2020 reported that pregnant women, regardless of their economic standing, were at risk of developing depression if they experienced COVID-19-associated financial strain [84]. These results were inconsistent with our study. The disparity between the studies may be due to differences in population characteristics, study settings, and methodologies used.

Assessing the mental health status of perinatal women at every stage of pregnancy and postpartum is highly important [85]. The detection of mental health disorders is enhanced by standardized screening [86]. Healthcare providers should design screening programs for early identification of mental health disorders in pregnant women and improve outcomes for the mother and her baby [87]. Studies have demonstrated that there was an association between symptoms of depression and anxiety and concern about threats of COVID-19 to the mother and baby’s life [50], and women with COVID-19 and pregnancy worries were more likely to meet the screening threshold for anxiety [88].

Additionally, e-screening can improve the efficiency of mental healthcare during emergencies and crises. China took extensive measures in this area, sets up mental health teams, and establish online or in-person mental health screening centers during the COVID-19 pandemic [89]. Due to the additional stressors resulting from the pandemic and unplanned modifications to birth plans, attention to perinatal mental health through telehealth screening is of pareamount importance [90].

## Strengths and limitations

This study has several strengths and limitations worth noting. This study was conducted during the fourth wave of COVID-19 in the country. Further, in this study, the mental health status of pregnant women was examined based on their socio-economic status. This study has also worth mentioning limitations. First, we did not consider the mental health status of pregnant women before the COVID-19 pandemic. Therefore, we were unable to compare pregnant women’s current mental health status (during the study period) with before the pandemic, and to identify pregnant women’s needs (e.g. mental health care services) in order to take actions to enhance their mental health status and well-being. Another limitation of this study was the limited access to pregnant women due to home quarantine and social restrictions. To overcome this, a multi-stage cluster sampling method was used. Also, we utilized a cross-sectional design, which does not warrant causal inference. Finally, the recruitment of participants from one of the counties of Iran implies that the findings may not be generalized to the whole pregnant women population of the country.

## Conclusion

According to the result, social dysfunction was the most common mental health problem among pregnant women. Practical strategies to promote social support and emotional coping can reduce stress and depressive symptoms. In order to protect pregnant women’s mental health and minimize the risks to women and child development, it is vital to provide pregnant women with appropriate psychological support during emergencies and crisis. Enough support should be explored as a priority during pregnancy and in post-birth in order to provide adequate support with mental health tied to early pregnancy, and high-risk pregnancies. Offering mental health support early enough to pregnant women can also reduce the negative effect of stressors on their mental and emotional well-being as well as on the growth and the development of their unborn babies. Moreover, maternal and child care centers should be aware of the demands of pregnant women, and provide adequate and accessible health education and service for the pregnant women’s safety and the infant. Health professionals can target pregnant women and improve their mental and emotional well-being.

Health policymakers, healthcare professionals, and obstetricians should emphasize addressing maternal mental well-being during and after global health crises. Maternal mental health should be one of the public health priorities during emergencies and crisis to enhance pregnant women’s well-being.

Future studies can explore the role of health professionals, home visitors, and online training classes to assist in the perinatal period and reduce risks for perinatal mental health problems during emergencies and crisis. Additionally, since we did not examine the role of healthcare services (e.g. maternity services, e-screening, and psychiatric tools for screening antenatal and perinatal mental disorders) in providing support to pregnant women, further studies are needed to examine the role of providing such services and health providers in improving the mental and emotional well-being of pregnant women during emergencies like COVID-19.

## Supporting information

S1 File STROBE statement—Checklist of items that should be included in reports of *cross-sectional studies*. (DOC)
